# Electroacupuncture enhances mesenchymal stem cell therapy via improved perfusion and inflammation modulation in peripheral nerve injury: an IVIM-MRI study in rats

**DOI:** 10.3389/fneur.2025.1642150

**Published:** 2025-08-08

**Authors:** Junfeng Li, Qiuyi Chen, Jintong Pan, Fanqi Meng, Wensheng Huang, Yingying Liang, Xuewen Yu, Ruirui Qi, Peiyin Luo, Haodong Qin, Yueyao Chen, Xiaofeng Lin

**Affiliations:** ^1^Department of Radiology, The Fourth Clinical Medical College of Guangzhou University of Chinese Medicine (Shenzhen Traditional Chinese Medicine Hospital), Shenzhen, China; ^2^Department of Nephrology, Shenzhen Hengsheng Hospital, Shenzhen, China; ^3^Department of Radiology, The Seventh Affiliated Hospital, Sun Yat-sen University, Shenzhen, China; ^4^Department of Pathology, The Fourth Clinical Medical College of Guangzhou University of Chinese Medicine (Shenzhen Traditional Chinese Medicine Hospital), Shenzhen, China; ^5^MR Research Collaboration, Siemens Healthineers, Shanghai, China; ^6^Department of Nuclear Medicine, The Seventh Affiliated Hospital, Sun Yat-sen University, Shenzhen, China

**Keywords:** peripheral nerve injuries, electroacupuncture, mesenchymal stem cells, microcirculation, magnetic resonance imaging, nerve regeneration

## Abstract

**Background:**

Stem cells are widely applied in peripheral nerve repair; however, their therapeutic potential is constrained by immune rejection, inflammatory responses, and a poor regenerative microenvironment. Therefore, reducing the inflammatory response, improving the regenerative environment and dynamically monitoring these processes by imaging techniques are critical. This study examined the effectiveness of electroacupuncture (EA) and bone mesenchymal stem cells (BMSCs) on acute sciatic nerve injury in rats. By employing intravoxel incoherent motion (IVIM) MRI, the study monitored perfusion and explored how EA improves the regenerative environment to optimize stem cell transplantation outcomes.

**Methods:**

Seventy-two rats were randomly assigned to four groups: EA, EA + BMSCs, BMSCs, and PBS. EA was applied at GB30 and ST36. IVIM-MRI (perfusion fraction f), T2WI, histological staining, immunostaining (CD31, IL-1α, IL-10, PPARγ), and SFI were used to evaluate treatment effects.

**Results:**

At 2–4 weeks, the nerve perfusion fraction f in the EA group recovered faster than in the BMSCs group (*p* < 0.05). By week 4, the EA group showed the greatest myelin regeneration and nerve fiber restoration (*p* < 0.05). The expression of vascular marker CD31 and anti-inflammatory markers IL-10 and PPARγ increased (*p* < 0.05), while pro-inflammatory marker IL-1α decreased in the EA and EA + BMSCs groups (*p* < 0.05). Furthermore, *f* values were strongly correlated with histological and functional outcomes (*p* < 0.05).

**Conclusion:**

EA is more effective than BMSCs alone in promoting nerve repair, enhancing blood flow, and reducing inflammation. Moreover, EA enhances the anti-inflammatory effects of BMSCs. The perfusion fraction (f) is a sensitive biomarker for evaluating nerve repair and perfusion restoration.

## 1 Introduction

Peripheral Nerve Injury (PNI) leads to enduring functional and physiological deficits in humans and animals (1, 2). Following PNI, maintaining an optimal inflammatory microenvironment is critical for the regeneration and repair of neural cells, as well as for the reconstruction of the microcirculatory system. This reconstruction supplies vital nutrients, enhances blood perfusion, and supports metabolism for nerve regeneration, while regulating the repair capacity of neural tissue through sophisticated biological processes (3, 4).

Bone mesenchymal stem cells (BMSCs) exhibit effectiveness in treating peripheral nerve damage, promoting nerve regeneration, and facilitating functional recovery, positioning them as a promising therapeutic approach with significant clinical potential for PNI (5, 6). However, the regenerative microenvironment significantly impacts the survival, growth, and specialization of transplanted stem cells. Research has indicated that the therapeutic efficacy of stem cell transplantation is often limited due to immune rejection-related inflammation and an unfavorable regenerative microenvironment (7). Consequently, promoting the reconstruction of the microcirculatory system and enhancing the regenerative microenvironment are critical for nerve regeneration and repair, representing an urgent challenge that must be addressed to enhance the outcomes of stem cell transplantation in PNI repair.

Traditional Chinese acupuncture provides distinct benefits in nerve repair, acting as a powerful method to stimulate nerve regrowth and improve functional restoration. Electroacupuncture (EA), by alleviating tissue edema, suppressing inflammatory responses, and exerting analgesic effects, has been demonstrated to provide substantial therapeutic benefits for PNI (8–10). Wang et al. further demonstrated that EA can enhance microcirculatory blood perfusion in areas affected by peripheral neuropathy, indicating its potential to promote nerve regeneration by improving local blood supply (11). However, current evaluations of EA’s efficacy in treating peripheral neuropathy predominantly rely on subjective functional scores, lacking quantitative and visualized reference standards (12). Therefore, this study aims to employ MRI techniques to quantitatively assess the therapeutic efficacy of EA.

Previous studies have commonly used electrophysiological methods to assess nerve regeneration; however, these techniques are invasive and do not allow for visual observation. Conventional MRI and diffusion tensor imaging (DTI) primarily reflect structural changes and axonal continuity but are limited in evaluating microcirculatory perfusion and water molecule diffusion (13). In this study, intravoxel incoherent motion MRI (IVIM-MRI) is employed, which simultaneously provides pathophysiological information related to blood perfusion and inflammation, and dynamically track nerve regeneration process. IVIM-MRI generates three parameters: f (perfusion fraction), D (diffusion coefficient), and D* (pseudo-diffusion coefficient). Among them, the *f* value, denoting the proportion of vascular and luminal fluid to total tissue fluid, acts as an indirect measure of tissue blood supply (14, 15). Recent successful applications of IVIM in stroke and muscular diseases underscore its potential utility in PNI (16, 17). Thus, this study utilizes IVIM to track microcirculation shifts dynamically during peripheral nerve repair, integrating histopathological markers (particularly inflammation-related factors), to investigate the role of EA in improving the regenerative microenvironment following stem cell transplantation. Additionally, this study compares the therapeutic efficacy of EA and BMSCs and proposes novel imaging biomarkers.

## 2 Materials and methods

### 2.1 Animals and surgical procedures

The research adhered strictly to the ethical standards and protocols for animal surgical procedures set forth by Guangzhou University of Chinese Medicine. Additionally, the study received formal approval from the university’s Institutional Animal Use and Care Committee (approval number: SCXK (Yue) 2023–0068, approval date: June 6, 2023). A total of 72 healthy adult male Sprague–Dawley rats (220 ± 20 g) were provided by the Experimental Animal Center of Jennio Biotech Co., Ltd. (Guangzhou, China). Throughout the study, every effort was made to minimize animal suffering and ensure their welfare. Animals were housed in standard laboratory cages under controlled environmental conditions: temperature (22–26°C), relative humidity (40–60%), and a 12-h light/dark cycle. Food and water were provided ad libitum. Postoperative analgesia was administered using buprenorphine (0.05 mg/kg, subcutaneously) every 12 h for 48 h to alleviate postoperative pain. Daily monitoring was conducted to assess signs of distress, infection, or functional impairment. Animals exhibiting severe or unrelieved distress were promptly and humanely euthanized in accordance with predefined humane endpoints. The study was conducted in accordance with the ARRIVE guidelines (Animal Research: Reporting of *In Vivo* Experiments).

The sciatic nerve injury model was established following the methodology described by Chen et al. (18). After intraperitoneal anesthesia with sodium pentobarbital (30 mg/kg), rats were secured in a prone position. In a meticulously controlled, sterile environment, the left sciatic nerve was carefully isolated, and a specialized hemostatic forceps (Shanghai Medical Instruments, China) was used to apply a constant pressure of 150 g to crush the midsection of the nerve trunk for 60 s. Under a stereomicroscope (Olympus BX60, Japan), it was confirmed that the nerve fascicles were completely severed, with the epineurium left undamaged. The contralateral nerve served as an autogenous control.

Postoperatively, the animals were randomly divided into four distinct groups: Group A (EA, *n* = 18), Group B (EA + BMSCs, *n* = 18), Group C (BMSCs, *n* = 18), and Group D (PBS, *n* = 18), which served as the control group. Each main group was further subdivided into two subsets: A1-D1 (*n* = 8 each) for functional evaluation and A2-D2 (*n* = 10 each) for histological examination. In SD rats, BMSCs were isolated and cultured following established protocols (18). Electroacupuncture (EA) treatment commenced on the second day post-surgery, with rats in Groups A and B receiving stimulation at the Huantiao (GB30) and Zusanli (ST36) acupoints (9, 19). Stem cell injections were administered intraoperatively: Groups B and C received a subcutaneous injection of 3 μL BMSCs suspension (5 × 10^5^ cells/μL) at the injury site, while Group D received an equivalent volume of phosphate-buffered saline (PBS).

**Figure 1:**
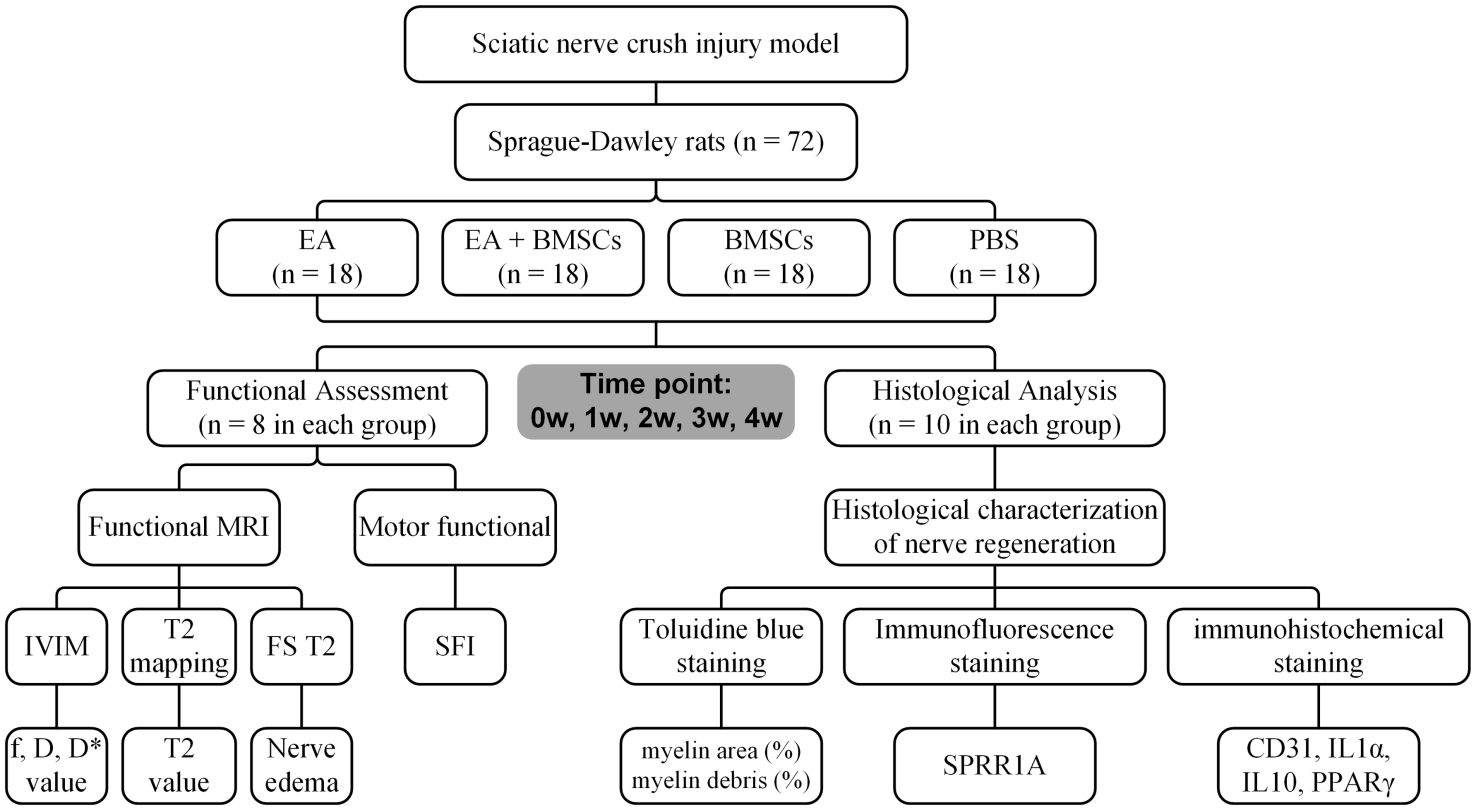
Study timeline and flowchart. W: week; EA, electroacupuncture; BMSCs, bone mesenchymal stem cells; PBS, phosphate-buffered saline; f, perfusion fraction; D, diffusion coefficient; D*, pseudo-diffusion coefficient; SFI, sciatic nerve function index.

To acquire longitudinal MRI data, the functional assessment subgroups (A1-D1) underwent MR imaging at baseline (week 0) prior to surgery and weekly from weeks 1 to 4 post-surgery. Concurrently, for the histological analysis subgroups (A2-D2), two rats were randomly selected and euthanized each week for tissue sampling, enabling evaluation at five time points in total. Figure 1 illustrates the research framework.

### 2.2 EA treatment

Beginning on the second day post-modeling, acupuncture needles were placed at the specified points on the affected side for both Group A and Group B. Groups C and D were similarly restrained but did not receive needle insertion. The G6805-C electroacupuncture device (Shanghai Huayi (Group) Co., Ltd., China) was used for acupoint stimulation, with the positive electrode attached to GB30 and the negative electrode to ST36. Stimulation parameters were set to a biphasic sparse-dense wave (alternating 2/100 Hz, intensity 1 mA), with the current adjusted to elicit slight muscle twitching in the affected limb. The treatment consisted of daily 20-min sessions, conducted 5 days a week, over a 4-week intervention period.

### 2.3 MR imaging

MRI scans were performed using a 3 T scanner (MAGNETOM Prisma, Siemens Healthcare, Erlangen, Germany). Sciatic nerve imaging was conducted preoperatively and at weeks 1, 2, 3, and 4. Rats were anesthetized prior to imaging to minimize motion and ensure scan quality.

We acquired IVIM-DWI, coronal T2-mapping, and coronal fat-suppressed T2-weighted imaging (FS-T2WI). Among these, IVIM-DWI was performed with a single-shot echo-planar imaging sequence. The parameters were as follows: repetition time = 3,500 ms, echo time = 72 ms, field of view (FOV) = 120 × 120 mm^2^, flip angle = 30°, slice thickness = 1.5 mm, slice gap = 0 mm, b-value = 0, 50, 100, 150, 200, 300, 400, 600, 800, and 1,000 s/mm^2^, and number of slices = 20, and scan time = 3 min 28 s. The coronal T2-mapping was obtained using a multi-slice, multi-echo spin-echo sequence. The parameters were as follows: repetition time = 1,400 ms, echo time = 17–80 ms, resolution = 256 × 256, FOV = 80 × 80 mm^2^, mean = 1, slice thickness = 1.0 mm, number of slices = 10, voxel size = 0.3 × 0.3 × 1.0 mm^3^.

### 2.4 Imaging analysis

The signal-to-noise ratio (SNR) of the IVIM-MRI images was preliminarily assessed during scanning by experienced radiologists through visual inspection. All included images met the diagnostic quality standards. IVIM images meeting the measurement criteria were post-processed using the Body Diffusion Toolbox on the Siemens Frontier platform to generate parametric maps for f, D, and D* (20). These maps were subsequently imported into the Siemens MRWP workstation, and measurements were conducted in the view interface. Two authors (Fanqi Meng and Wensheng Huang, each with 10 years of experience in the radiology department), blinded to the study groups and working independently, performed the measurements. The sciatic nerve’s position was pinpointed using T2WI, and specific rectangular regions of interest (ROIs), each spanning 40 pixels, were selected to assess the f, D, and D* values. These measurements were taken from both the near and far sections of the injured sciatic nerve, as well as from the adjacent muscle tissue. Each ROI was delineated three times per region, and the mean values were calculated to determine the final f, D, and D* values.

Meanwhile, to assess nerve edema degree, the two authors (Fanqi Meng and Wensheng Huang, each with 10 years of experience in the radiology department) first outlined the ROIs on the T2 map as described above and then measured the T2 relaxation time.

Table 1 summarizes the MRI sequences utilized in this study, along with the associated pathophysiological information provided by these sequences.

**Table 1:** MRI sequences used in this study and the information they reflect.

Sequence	Quantitative metric	Information reflected
Coronal FS-T2WI	––––	Tissue structure, nerve edema degree
Coronal T2-mapping	Tissue relaxation time (T2 value)	Quantification of nerve edema
IVIM-DWI	Perfusion fraction (f) Diffusion coefficient (D) Pseudo-diffusion coefficient (D*)	Microcirculatory blood perfusion

### 2.5 Motor functional assessment

Gait analysis was performed in rats from Groups A1-D1 at each MRI time point. Following the approach described by Chen et al., the sciatic functional index (SFI) was calculated to quantitatively assess motor dysfunction (18).

### 2.6 Histology

At predetermined intervals (weeks 0, 1, 2, 3, and 4), magnetic resonance imaging was conducted, after which two rats from the A2-D2 subgroups were randomly selected and humanely euthanized via cardiac perfusion using a PBS solution infused with 4% paraformaldehyde.

Toluidine blue staining was performed to analyze the structure of myelin sheaths, and SPRR1A immunofluorescence staining was used to assess axonal regeneration. The specific staining procedures and quantitative analyses were conducted with reference to the study by Chen et al. (13).

Immunohistochemistry for CD31 (MAB-0720, MXB Biotechnologies), IL-1α (ab239517, Abcam), IL-10 (AF519, R&D), and PPARγ (ab41928, Abcam) was performed on 4 μm paraffin sections using the streptavidin-biotin-peroxidase method. Antigen retrieval was carried out in 98°C citrate buffer, followed by peroxidase blocking, serum blocking, and overnight primary antibody incubation. After exposure to secondary antibodies and streptavidin-peroxidase, diaminobenzidine (DAB) was used for chromogenic detection, and sections were counterstained with hematoxylin (21). For quantitative analysis, digital whole-slide images were acquired using the PANNORAMIC SCAN system (3DHISTECH Ltd., Hungary). Three random fields per image were analyzed via ImageJ for the percentage of positive staining area using color deconvolution and thresholding (22).

Transmission electron microscopy (TEM) was used to observe the microstructural changes of myelin sheath during nerve regeneration (23).

### 2.7 Statistical analysis

Each group initially included 10 rats. Rats that died during follow-up or had poor-quality MRI images were excluded. Ultimately, 8 rats per group completed imaging at all scheduled time points (weeks 0, 1, 2, 3, and 4) and were included in the final statistical analysis. The Shapiro–Wilk test was used to confirm that the data were normally distributed. Data are presented as mean ± SD. MRI metrics (T2 value, f, D, and D*) were analyzed using mixed models, with time, group, and time × group included as fixed effects, and subject as a random effect. Effect sizes were estimated using partial eta squared (partial *η*^2^) (24). For histological and functional outcomes (myelin area, myelin debris area, CD31, IL-1α, PPARγ, IL-10, and SFI), one-way ANOVA was conducted. Bonferroni post-hoc correction was applied for multiple comparisons. Pearson coefficients were calculated to assess the relationships between IVIM and histopathological measures. Statistical analyses were performed using SPSS (version 25.0). Statistical significance was defined as *p* < 0.05.

To assess inter-observer reproducibility, the intraclass correlation coefficient (ICC) was calculated based on the IVIM parameters (f, D, and D*) measured by two radiologists. The ICC criteria were as follows: 0.5 ≤ ICC < 0.75, 0.75 ≤ ICC < 0.9, and ICC ≥ 0.9 indicate moderate, good, and excellent reproducibility, respectively (25).

## 3 Results

### 3.1 MRI for monitoring PNI repair in rats

#### 3.1.1 T2WI and T2 mapping of sciatic nerve injury and regeneration

**Figure 2:**
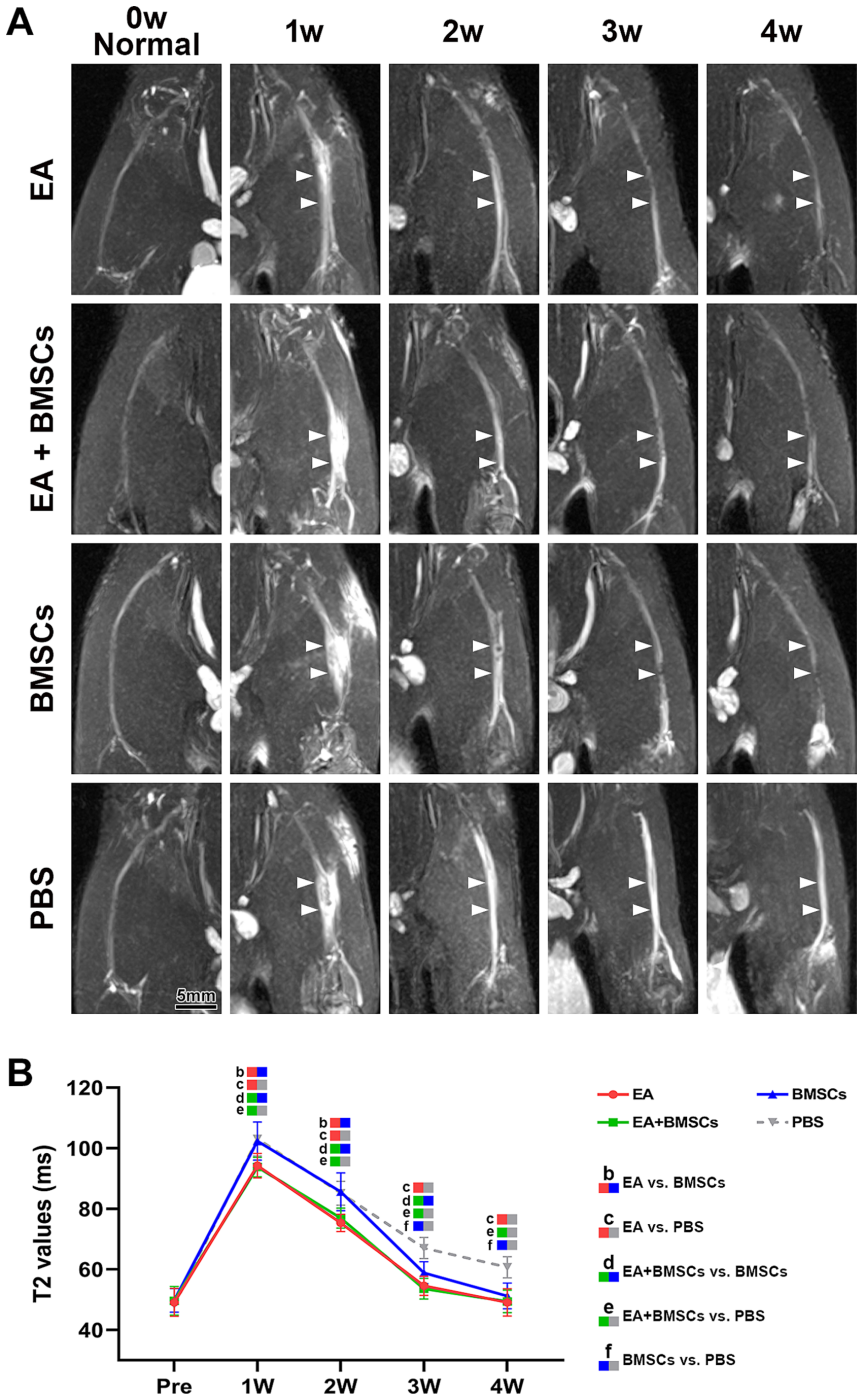
FS-T2WI of injured sciatic nerve and uninjured contralateral nerve. **(A)** At week 1 post-surgery, T2WI showed hyperintensity in the injured sciatic nerve across all groups due to edema, accompanied by nerve thickening and increased signal intensity. From weeks 2 to 4, nerve swelling (white arrows) and T2WI hyperintensity gradually decreased in all groups except the PBS group, returning to preoperative levels. Intergroup comparisons showed that at week 1, muscle and nerve edema were more pronounced in the BMSCs and PBS groups than in the EA and EA + BMSCs groups. By week 4, the PBS group still had mild residual edema, whereas the nerves in the other groups had largely returned to normal. **(B)** T2 values of the injured nerve peaked at week 1 and gradually returned to preoperative levels from weeks 2 to 4. Intergroup comparisons revealed that the EA (red) and EA + BMSCs (green) groups, both receiving electroacupuncture, consistently exhibited lower T2 values than the PBS group (gray). In the BMSCs group (blue), T2 values showed no significant difference from the PBS group in the early stages (weeks 1–2), but became lower than the PBS group at weeks 3–4. Table 2 shows the specific statistical analysis. Scale bar = 5 mm; W: week; EA, electroacupuncture; BMSCs, bone mesenchymal stem cells; PBS, phosphate-buffered saline.

Fat-saturated T2-weighted imaging (FS-T2WI) was utilized to visualize the morphological and edema changes of the injured sciatic nerve recovery in each group (Figure 2A). T2 mapping was utilized to measure the T2 value, which could quantify the degree of nerve edema. Mixed model analysis revealed significant main effects of time and group, as well as a significant time × group interaction for the T2 values of the injured nerves (all *p* < 0.001; partial *η*^2^ = 0.968, 0.785 and 0.337, respectively). The T2 values of injured nerves increased rapidly within the first week post-surgery and gradually recovered from weeks 2 to 4 (Figure 2B). Intergroup comparisons revealed that at 1–2 weeks, the T2 values in the EA and EA + BMSCs groups, both receiving electroacupuncture treatment, were significantly lower than those in the BMSCs and PBS groups (EA vs. BMSCs: *p* = 0.017, <0.001; EA vs. PBS: *p* = 0.008, 0.001; EA + BMSCs vs. BMSCs: *p* = 0.011, 0.002; EA + BMSCs vs. PBS: *p* = 0.005, 0.004). By weeks 3–4, T2 values were significantly lower in the BMSCs group than in the PBS group (all *p* < 0.001; Table 2).

**Table 2:** Injured sciatic nerves T2 and IVIM parameters in EA, EA + BMSCs, BMSCs and PBS (mean ± SD).

MRI	Time points (weeks)	EA	EA + BMSCs	BMSCs	PBS	*p* < 0.05
T2	0	49.10 ± 4.61	49.63 ± 4.74	49.75 ± 3.92	48.23 ± 2.35	–
	1	94.24 ± 4.01	93.79 ± 3.23	102.34 ± 6.32	103.05 ± 5.60	bcde
	2	75.33 ± 2.84	76.90 ± 3.30	85.60 ± 6.24	85.13 ± 3.94	bcde
	3	54.56 ± 3.13	53.61 ± 3.42	58.85 ± 3.65	67.04 ± 3.50	cdef
	4	49.11 ± 4.49	49.41 ± 3.73	51.20 ± 4.24	60.68 ± 3.50	cef
f	0	0.0454 ± 0.0027	0.0460 ± 0.0035	0.0455 ± 0.0038	0.0455 ± 0.0044	–
	1	0.0164 ± 0.0017	0.0167 ± 0.0020	0.0156 ± 0.0018	0.0072 ± 0.0024	cef
	2	0.0296 ± 0.0065	0.0258 ± 0.0027	0.0216 ± 0.0073	0.0127 ± 0.0023	bcef
	3	0.0264 ± 0.0046	0.0236 ± 0.0026	0.0213 ± 0.0031	0.0148 ± 0.0021	bcef
	4	0.0374 ± 0.0047	0.0349 ± 0.0021	0.0316 ± 0.0037	0.0225 ± 0.0035	bcef
D	0	0.0014 ± 0.0002	0.0012 ± 0.0001	0.0013 ± 0.0001	0.0012 ± 0.0001	–
	1	0.0014 ± 0.0002	0.0014 ± 0.0005	0.0013 ± 0.0001	0.0016 ± 0.0005	–
	2	0.0016 ± 0.0005	0.0013 ± 0.0002	0.0012 ± 0.0001	0.0013 ± 0.0001	–
	3	0.0013 ± 0.0003	0.0012 ± 0.0002	0.0013 ± 0.0003	0.0014 ± 0.0004	–
	4	0.0013 ± 0.0001	0.0011 ± 0.0001	0.0011 ± 0.0001	0.0011 ± 0.0001	–
D*	0	0.0550 ± 0.0590	0.0748 ± 0.0613	0.0396 ± 0.0448	0.0284 ± 0.0154	–
	1	0.0310 ± 0.0477	0.0148 ± 0.0027	0.0135 ± 0.0030	0.0101 ± 0.0015	–
	2	0.0142 ± 0.0022	0.0438 ± 0.0442	0.0494 ± 0.0612	0.0299 ± 0.0480	–
	3	0.0720 ± 0.0646	0.0340 ± 0.0464	0.0321 ± 0.0471	0.0368 ± 0.0455	–
	4	0.0250 ± 0.0119	0.0399 ± 0.0449	0.0721 ± 0.0636	0.0451 ± 0.0439	–

EA, electroacupuncture; BMSCs, bone mesenchymal stem cells; PBS, phosphate-buffered saline; SD, standard deviation; *p* ≤ 0.05, post-hoc analysis with Bonferroni correction; f, perfusion fraction; D, diffusion coefficient; D*, pseudo-diffusion coefficient. *p* < 0.05 and statistically significant difference between groups: b, EA vs. BMSCs; c, EA vs. PBS; d, EA + BMSCs vs. BMSCs; e, EA + BMSCs vs. PBS; f, BMSCs vs. PBS.

#### 3.1.2 IVIM MRI for injured sciatic nerve perfusion assessment

**Figure 3:**
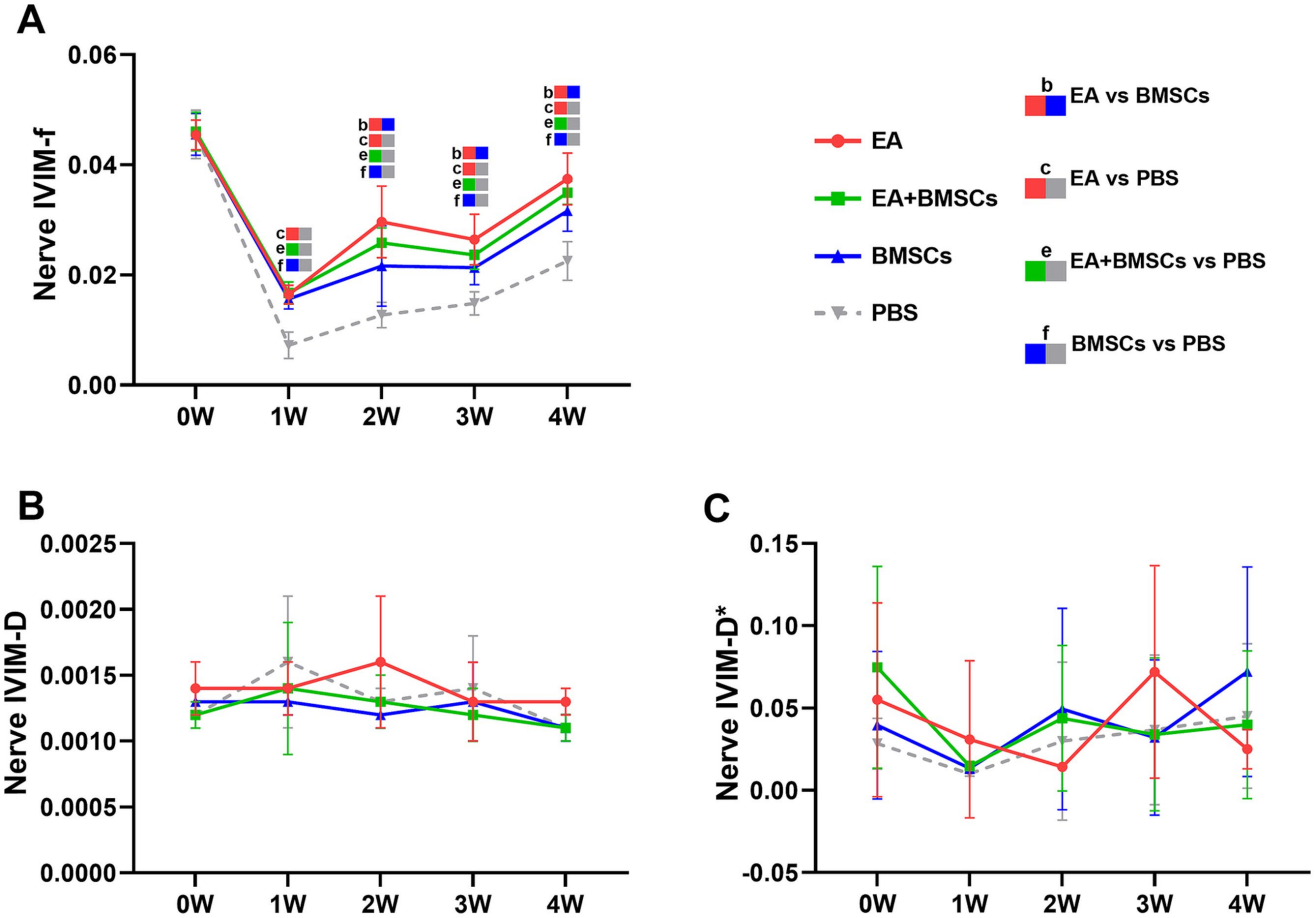
IVIM of the injured sciatic nerve in rats. At 4 weeks post-surgery, the f, D, and D* values of all injured sciatic nerves were compared with their respective control values. **(A)** At week 1 post-surgery, the nerve *f* values in all groups decreased; from weeks 2 to 4, the nerve f values gradually recovered, with the EA group (red) demonstrating a faster recovery rate than the other groups, indicating that nerve perfusion in the EA group recovered most rapidly. **(B,C)** No distinct patterns were observed in the D and D* values across the groups. Table 2 shows the specific statistical analyses. W: week; IVIM, Intravoxel Incoherent Motion; EA, electroacupuncture; BMSCs, bone mesenchymal stem cells; PBS, phosphate-buffered saline; f, perfusion fraction; D, diffusion coefficient; D*, pseudo-diffusion coefficient.

The ICCs for the measurements of f, D, and D* by the two radiologists were 0.856 (95% CI: 0.525–0.962), 0.526 (95% CI: −0.111–0.857), and 0.232 (95% CI: −0.430–0.732), respectively. Mixed model analysis revealed significant main effects of time and group, as well as a significant time × group interaction for the perfusion fraction (f) of the injured nerves (all *p* < 0.001; partial *η*^2^ = 0.939, 0.788 and 0.401, respectively). From weeks 1 to 4 following nerve injury, the *f* values in all groups initially declined and then gradually increased, which indicated recovery of perfusion in the injured nerve post-treatment (Figure 3A). From weeks 2 to 4, there were significant differences in f values among the treatment groups and the control group (all *p* < 0.05). Among the treatment groups, the f values were ranked in the following order: EA group > EA + BMSCs group > BMSCs group (EA vs. EA + BMSCs: *p* = 0.945, 0.570, 1.000; EA vs. BMSCs: *p* = 0.027, 0.024, 0.020; EA + BMSCs vs. BMSCs: *p* = 0.685, 1.000, 0.462), suggesting that nerve perfusion recovery was greater in the EA group than in the remaining treatment groups (Table 2).

Statistical analysis of the D value and the D* value did not reveal clear patterns of change (Figures 3B,C).

#### 3.1.3 IVIM MRI for muscle perfusion assessment

**Figure 4:**
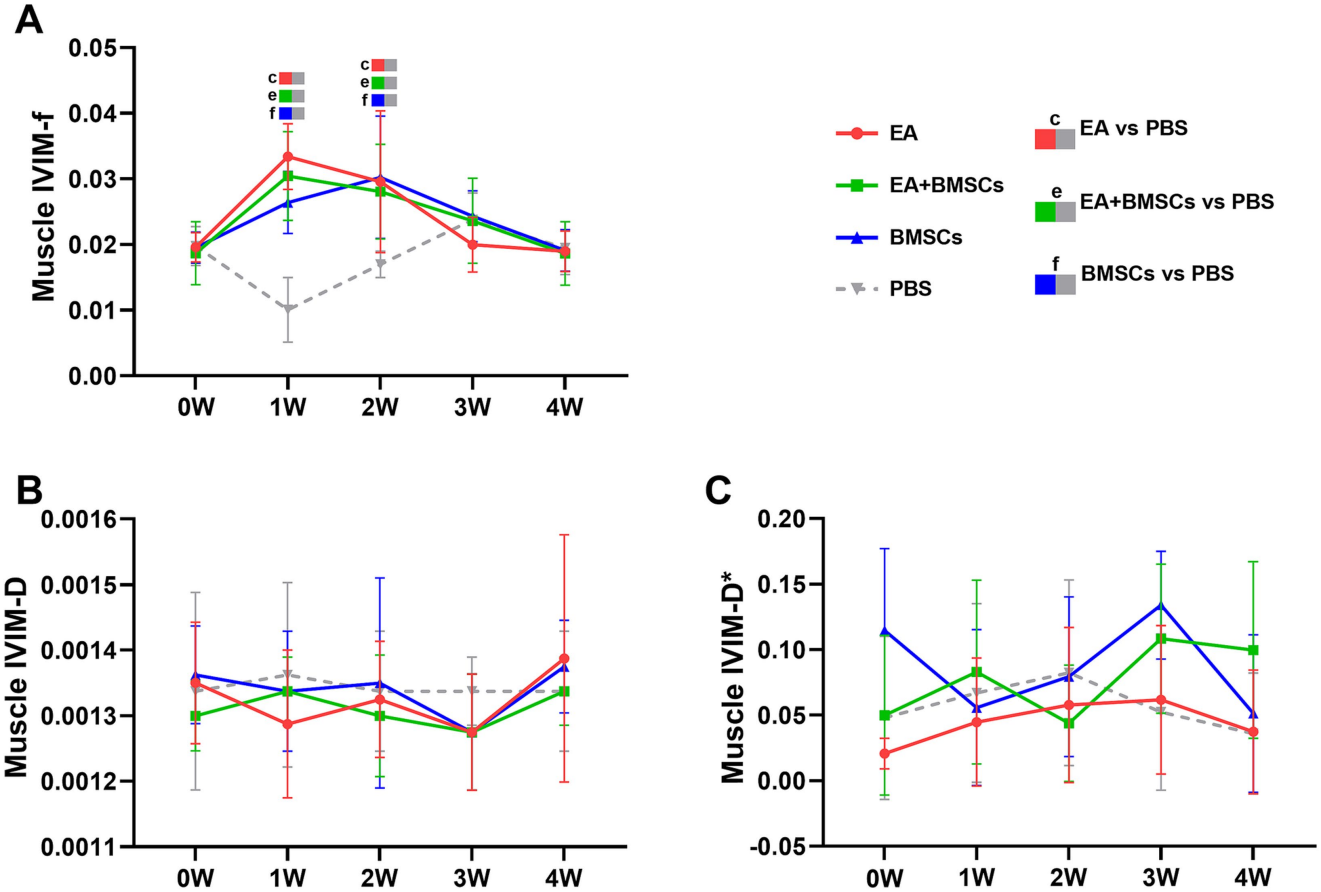
IVIM of the surrounding muscles of the injured sciatic nerve in rats. Post-surgery, the f, D, and D* values of the muscles surrounding all injured nerves were compared with their respective control values. **(A)** The f values in the three treatment groups exhibited an overall trend of initial increase followed by a decline. In the EA group (red) and EA + BMSCs group (green), both receiving electroacupuncture, muscle f values peaked at week 1, whereas in the standalone BMSCs group (blue), the peak occurred at week 2. **(B,C)** No clear patterns were observed in the D and D* values across the groups. Table 3 shows the specific statistical analysis. W: week; IVIM, Intravoxel Incoherent Motion; EA, electroacupuncture; BMSCs, bone mesenchymal stem cells; PBS, phosphate-buffered saline; f, perfusion fraction; D, diffusion coefficient; D*, pseudo-diffusion coefficient.

Mixed model analysis revealed a significant main effect of group on the perfusion fraction (f) of the muscle adjacent to the injured nerve (*p* = 0.013; partial *η*^2^ = 0.282), while the effects of time and the time × group interaction were not significant (*p* = 0.882 and 0.821, respectively). From weeks 1 to 4, the *f* values of muscles near the injured nerve in all treatment groups initially increased and then returned to baseline levels (Figure 4A). In contrast, the f values in the PBS group remained consistently low, indicating that blood perfusion in the surrounding muscles improved following treatment. Among the treatment groups, the EA group demonstrated the fastest increase in f values, followed by the EA + BMSCs group, with both reaching peak values at week 1 before gradually declining and stabilizing near normal levels by week 4. The BMSCs group showed a slower increase, peaking at week 2 before decreasing. During the early treatment phase (weeks 1–2), f values in all treatment groups were significantly higher than those in the PBS group (all *p* < 0.05); however, in the later phase (weeks 3–4), no significant differences were observed among the treatment groups and the PBS group (Table 3).

**Table 3:** Muscle IVIM parameters in EA, EA + BMSCs, BMSCs and PBS (mean ± SD).

IVIM-MRI	Time points (weeks)	EA	EA + BMSCs	BMSCs	PBS	*p* < 0.05
f	0	0.0196 ± 0.0022	0.0187 ± 0.0048	0.0195 ± 0.0024	0.0198 ± 0.0030	–
	1	0.0334 ± 0.0050	0.0304 ± 0.0067	0.0264 ± 0.0047	0.0101 ± 0.0049	cef
	2	0.0296 ± 0.0108	0.0281 ± 0.0072	0.0303 ± 0.0093	0.0148 ± 0.0019	cef
	3	0.0200 ± 0.0042	0.0236 ± 0.0065	0.0243 ± 0.0039	0.0238 ± 0.0041	–
	4	0.0190 ± 0.0031	0.0186 ± 0.0049	0.0191 ± 0.0032	0.0195 ± 0.0040	–
D	0	0.0014 ± 0.0001	0.0013 ± 0.0000	0.0014 ± 0.0001	0.0013 ± 0.0002	–
	1	0.0013 ± 0.0001	0.0013 ± 0.0000	0.0013 ± 0.0001	0.0014 ± 0.0001	–
	2	0.0013 ± 0.0001	0.0013 ± 0.0001	0.0013 ± 0.0001	0.0013 ± 0.0001	–
	3	0.0013 ± 0.0001	0.0013 ± 0.0001	0.0013 ± 0.0001	0.0013 ± 0.0000	–
	4	0.0014 ± 0.0002	0.0013 ± 0.0000	0.0014 ± 0.0001	0.0013 ± 0.0001	–
D*	0	0.0207 ± 0.0116	0.0500 ± 0.0609	0.1148 ± 0.0624	0.0479 ± 0.0622	–
	1	0.0448 ± 0.0489	0.0830 ± 0.0701	0.0558 ± 0.0595	0.0669 ± 0.0682	–
	2	0.0578 ± 0.0592	0.0438 ± 0.0443	0.0794 ± 0.0609	0.0824 ± 0.0707	–
	3	0.0617 ± 0.0567	0.1085 ± 0.0569	0.1340 ± 0.0411	0.0524 ± 0.0597	–
	4	0.0373 ± 0.0473	0.0997 ± 0.0674	0.0513 ± 0.0601	0.0360 ± 0.0462	–

EA, electroacupuncture; BMSCs, bone mesenchymal stem cells; PBS, phosphate-buffered saline; SD, standard deviation; *p* ≤ 0.05, post-hoc analysis with Bonferroni correction; f, perfusion fraction; D, diffusion coefficient; D*, pseudo-diffusion coefficient. *p* < 0.05 and statistically significant difference between groups: c, EA vs. PBS; e, EA + BMSCs vs. PBS; f, BMSCs vs. PBS.

Statistical analysis of the D value and the D* value did not reveal clear patterns of change (Figures 4B,C).

### 3.2 Histologic features of neural repair

#### 3.2.1 Myelin regeneration assessed by toluidine blue staining and transmission electron microscopy (TEM)

**Figure 5:**
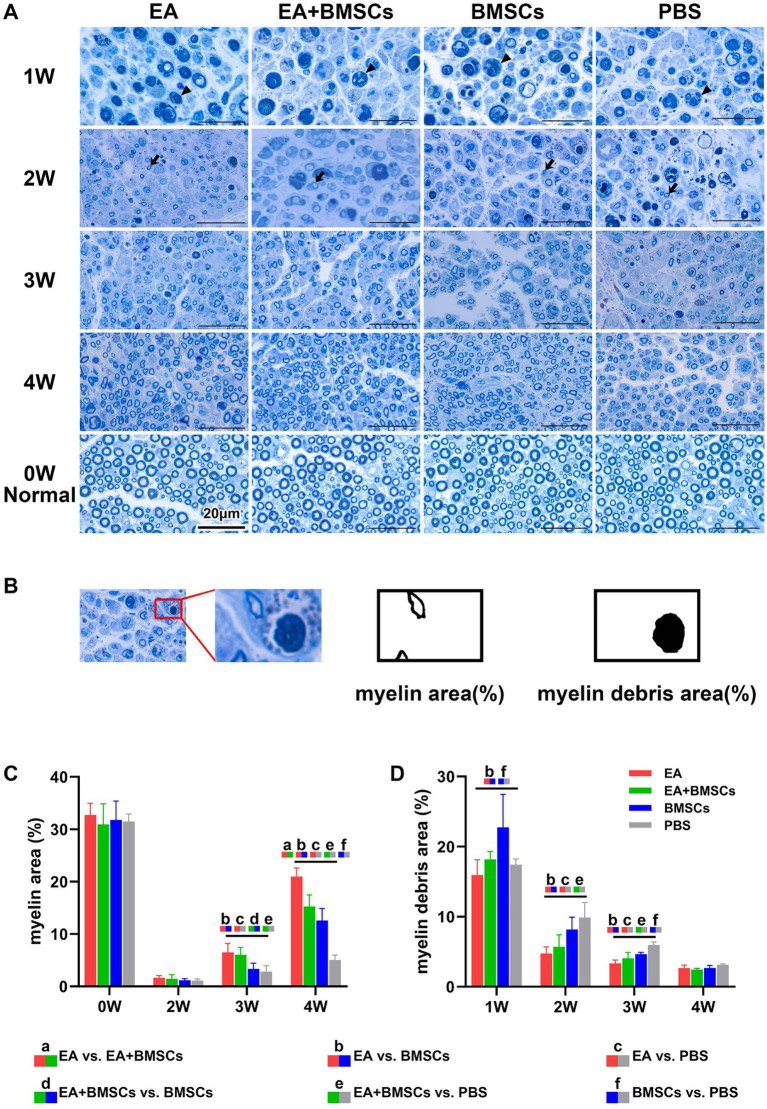
Toluidine blue staining of the injured and uninjured contralateral nerves. **(A)** Toluidine blue staining of injured and contralateral uninjured sciatic nerves. At week 1 post-surgery, all groups exhibited extensive macrophage infiltration engulfing myelin debris (triangular arrows). By week 2, myelin debris was mostly cleared in the EA group, while the EA + BMSCs group exhibited a few residual debris and macrophages. In contrast, the BMSCs and PBS groups retained more myelin debris and macrophages. Meanwhile, newly formed myelin sheaths with small thin-walled rings can be seen (dovetail arrows). At week 3, regenerated myelin area was significantly greater in the EA and EA + BMSCs groups than in the BMSCs and PBS groups. By week 4, myelin regeneration in the EA group were nearly complete, with increased myelin diameter and thickened walls. **(B)** Schematic representation of semi-quantitative analysis for myelin area and myelin debris area. **(C,D)** Comparison of the percentage of myelin area and myelin debris area in the injured nerves of each group with the contralateral uninjured nerve (control) post-surgery. At week 3, the myelin debris area was lower in the EA group (red) than in the other groups **(D)**. At weeks 3 and 4, the regenerated myelin area in the EA group (red) surpassed that of the other groups **(C)**. Table 4 shows the specific statistical analysis. Scale bar = 20 μm; W: week; EA, electroacupuncture; BMSCs, bone mesenchymal stem cells; PBS, phosphate-buffered saline.

Figures 5A,B illustrates toluidine blue staining of the injured sciatic nerve’s distal stump in each group. Semi-quantitative analysis of the regenerated myelin area percentage was performed (Figure 5C; Table 4). The findings revealed no significant differences in myelin area across groups before surgery or by week 2 post-operation. By week 3, the newly formed myelin area in the EA and EA + BMSCs groups markedly exceeded that in the BMSCs and PBS groups. (*p* = 0.011, 0.003, 0.035, and 0.010). At week 4, the EA group exhibited the highest regenerated myelin area (*p* = 0.001, <0.001, and <0.001), followed by the EA + BMSCs and BMSCs groups, indicating that EA treatment was the most effective in promoting myelin regeneration.

**Table 4:** Histological characterizations in EA, EA + BMSCs, BMSCs, and PBS (mean ± SD).

Histology of the injured nerves	Time points (weeks)	EA	EA + BMSCs	BMSCs	PBS	*p* < 0.05
Myelin area (%)	0	32.72 ± 2.25	30.93 ± 3.91	31.79 ± 3.58	31.48 ± 1.43	–
	2	1.66 ± 0.43	1.45 ± 0.78	1.18 ± 0.31	1.11 ± 0.31	–
	3	6.53 ± 1.67	6.05 ± 1.41	3.35 ± 1.09	2.82 ± 1.12	bcde
	4	20.97 ± 1.67	15.26 ± 2.22	12.56 ± 2.30	5.04 ± 0.92	abcef
Myelin debris area (%)	1	15.94 ± 2.18	18.18 ± 1.12	22.74 ± 4.69	17.43 ± 0.79	bf
	2	4.74 ± 0.94	5.70 ± 1.71	8.17 ± 1.76	9.88 ± 2.13	bce
	3	3.35 ± 0.47	4.05 ± 0.84	4.65 ± 0.26	5.95 ± 0.43	bcef
	4	2.67 ± 0.40	2.47 ± 0.17	2.68 ± 0.35	3.14 ± 0.14	–

EA, electroacupuncture; BMSCs, bone mesenchymal stem cells; PBS, phosphate-buffered saline; SD, standard deviation; *p* ≤ 0.05, post-hoc analysis with Bonferroni correction. *p* < 0.05 and statistically significant difference between groups: a, EA vs. EA + BMSCs; b, EA vs. BMSCs; c, EA vs. PBS; d, EA + BMSCs vs. BMSCs; e, EA + BMSCs vs. PBS; f, BMSCs vs. PBS.

**Figure 6:**
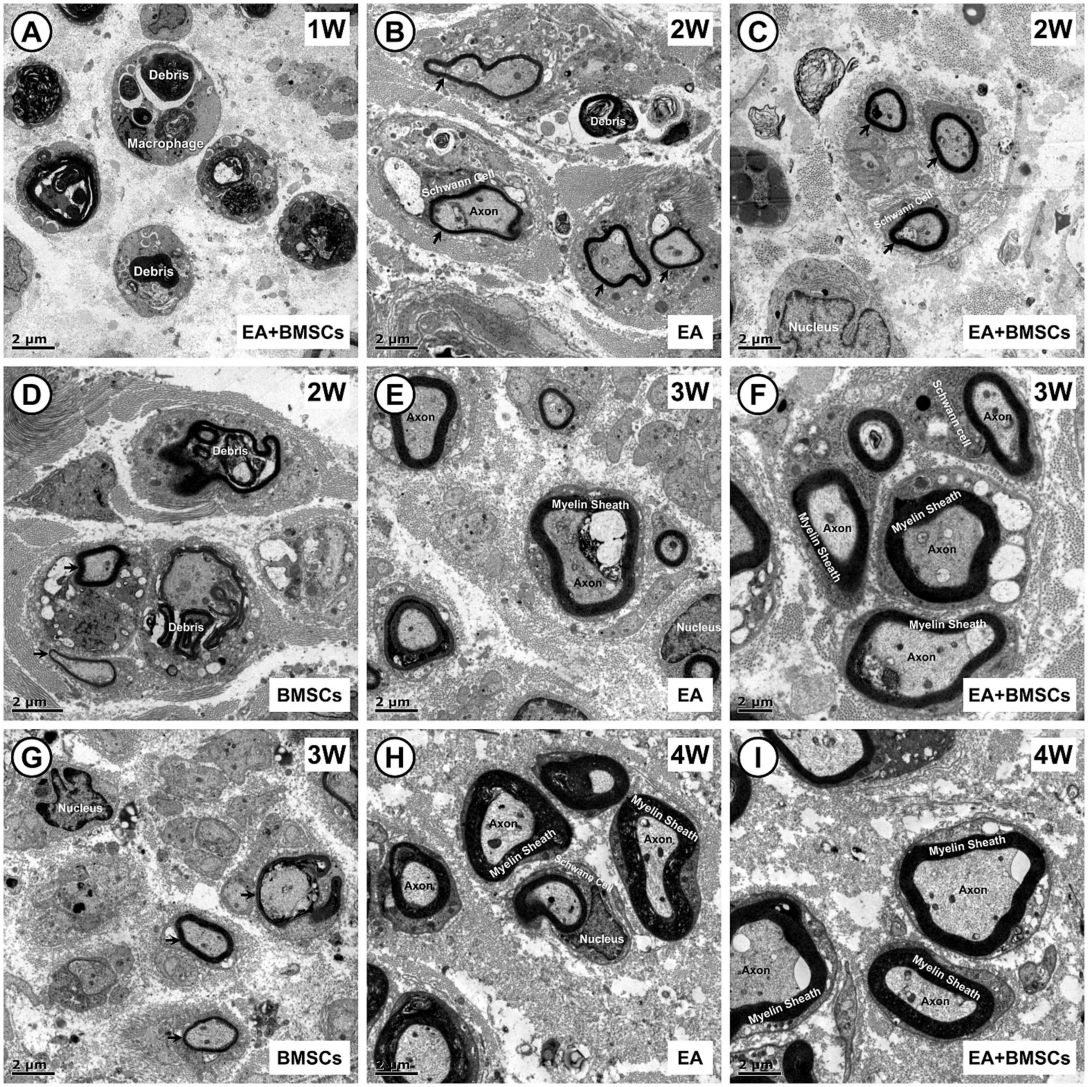
Transmission electron microscopic images of the growth process of the injured sciatic nerve. **(A)** In the first week post-surgery, nerve injury leads to myelin sheath splitting, wrinkling, and fragmentation, with debris phagocytosed by macrophages for degradation. **(B–D)** By week 2, myelin debris is largely cleared in the EA **(B)** and EA + BMSCs **(C)** groups, where Schwann cells proliferate actively, wrapping axons to form thin, small-diameter myelin sheaths (arrows). In contrast, the BMSCs group **(D)** shows fewer newly formed sheaths, with macrophages still containing incompletely degraded myelin fragments. **(E–G)** At week 3, myelin sheaths in the EA **(E)** and EA + BMSCs **(F)** groups continue to grow, displaying a regular morphology and thickened walls. In contrast, in the BMSCs group **(G)**, fewer myelin sheaths form, and their walls remain thin. **(H,I)** By week 4, axons and myelin sheaths in the EA **(H)** and EA + BMSCs **(I)** groups have nearly recovered, with structure and density approaching normal nerve fibers. Scale bars = 2 μm; W: week; EA, electroacupuncture; BMSCs, bone mesenchymal stem cells.

In addition, semi-quantitative analysis of the myelin debris area percentage was conducted (Figure 5D; Table 4). At week 1, the myelin debris area was significantly larger in the BMSCs group than in the EA and PBS groups (*p* = 0.006 and 0.038). At weeks 2 and 3, the myelin debris area was significantly smaller in the EA group than in the BMSCs and PBS groups (*p* = 0.033, 0.001, 0.010, and <0.001), suggesting that EA treatment was the most effective in facilitating debris clearance. By the fourth week, no notable disparities in the area of myelin debris were detected across the groups. TEM showed ultrastructural changes during sciatic nerve repair (Figure 6).

#### 3.2.2 SPRR1A immunofluorescence staining for assessing axonal regeneration

**Figure 7:**
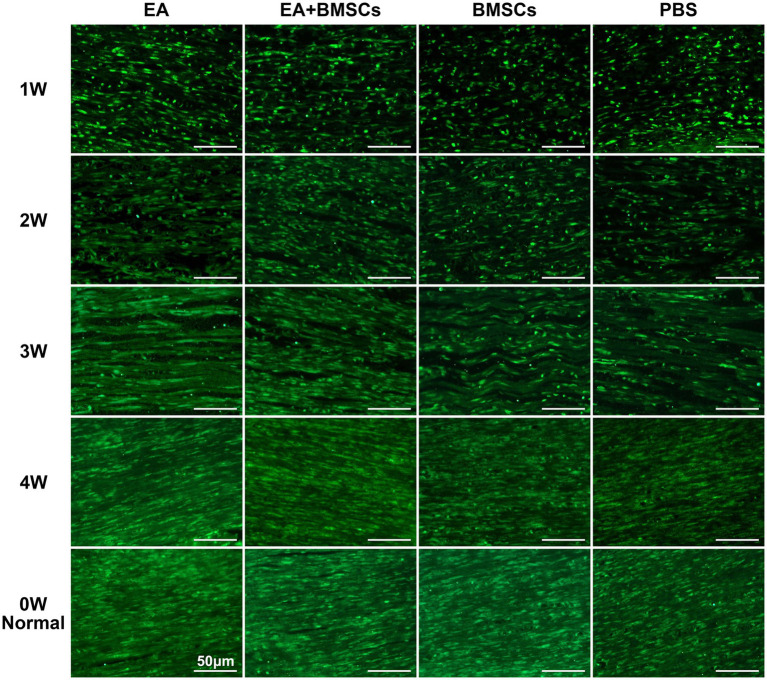
SPRR1A staining of the injured sciatic nerve. SPRR1A immunofluorescence staining, observed under a fluorescence microscope (400×), revealed the continuity of recovery in the injured sciatic nerve. Nerve regeneration and reconnection were significantly better in the EA group than in the PBS and BMSCs groups. Scale bar = 50 μm; W: week; EA, electroacupuncture; BMSCs, bone mesenchymal stem cells; PBS, phosphate-buffered saline.

The results of SPRR1A immunofluorescence staining for axons (Figure 7) revealed the following: At week 1 following acute sciatic nerve crush injury, nerve fiber disruption and reduced continuity were observed across all groups. By the second week, a gradual healing process had begun in all experimental groups, marked by the lengthening of axons. By week 3, the integrity of the damaged nerve fibers had improved noticeably, and clear differences began to emerge between groups. By week 4, the EA and EA + BMSCs groups showed near-complete restoration of nerve fiber structure, whereas the BMSCs and PBS groups exhibited delayed regeneration. Overall, the EA and EA + BMSCs groups exhibited the fastest nerve fiber recovery, followed by the BMSCs group, while the PBS group showed the slowest recovery.

#### 3.2.3 Immunohistochemical staining for assessing levels of CD31, IL-1α, IL-10 and PPARγ

**Figure 8:**
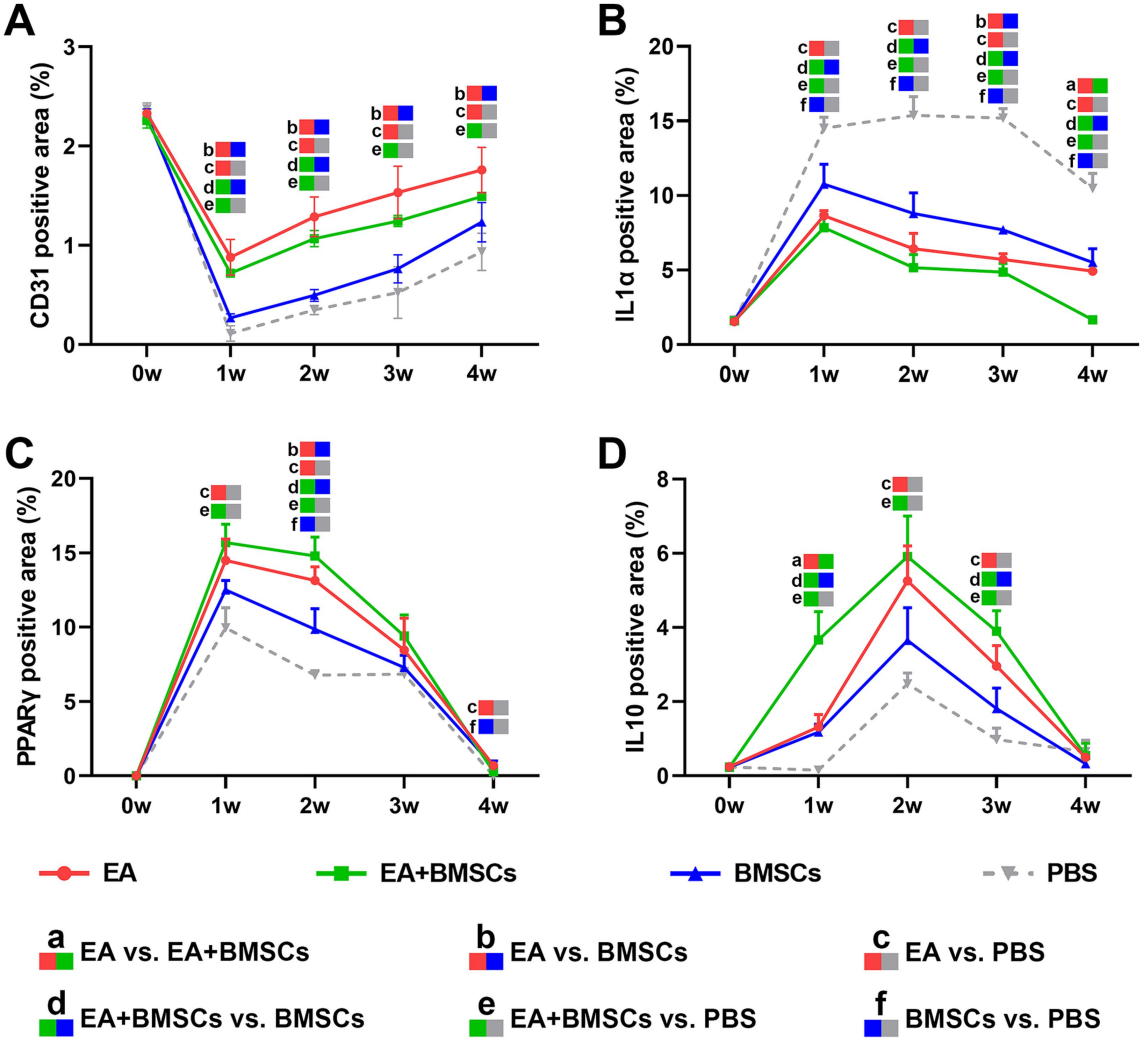
Expression of CD31 and inflammatory factors in the injured sciatic nerve of rats. **(A)** The temporal trends of vascular endothelial cell marker CD31 across groups were as follows: the expression of CD31 decreased in week 1 and gradually recovered in weeks 2–4. CD31 expression in the EA group was consistently higher than that in the BMSCs and PBS groups during weeks 1–4. CD31 expression in the EA + BMSCs group was higher than that in the BMSCs group and the PBS group during weeks 1–2. **(B-D)** The temporal trends of inflammatory cytokines across groups were as follows: IL-1α **(B)** and PPARγ **(C)** peaked at week 1 and subsequently declined, while IL-10 **(D)** peaked at week 2 before gradually decreasing. By week 4, all three inflammatory markers had returned to normal levels. At weeks 2 and 3, IL-1α **(B)** expression was lower in the EA + BMSCs group (green) than in the other groups, while PPARγ **(C)** and IL-10 **(D)** expression was higher. Table 5 shows the specific statistical analysis. W: week; EA, electroacupuncture; BMSCs, bone mesenchymal stem cells; PBS, phosphate-buffered saline.

CD31 is a marker of vascular endothelial cells, with immunohistochemical staining shown in Figure 8A. CD31 expression decreased at week 1 after nerve injury but gradually increased from weeks 2 to 4, indicating progressive angiogenesis and improvement of microcirculation during the repair process. Comparison between groups showed that CD31 expression in the EA group was consistently higher than that in the BMSCs and PBS groups during weeks 1–4 (all *p* < 0.05). CD31 expression in the EA + BMSCs group was higher than that in the BMSCs group and the PBS group during weeks 1–2 (all *p* < 0.05; Table 5).

**Table 5:** Expression of vascular endothelial cell marker and inflammatory factors in EA, EA + BMSCs, BMSCs, and PBS (mean ± SD).

Inflammatory factor	Time points (weeks)	EA	EA + BMSCs	BMSCs	PBS	*p* < 0.05
CD31 positive area (%)	0	2.33 ± 0.03	2.26 ± 0.07	2.30 ± 0.07	2.37 ± 0.06	–
	1	0.88 ± 0.18	0.72 ± 0.04	0.27 ± 0.04	0.11 ± 0.08	bcde
	2	1.29 ± 0.20	1.07 ± 0.08	0.50 ± 0.06	0.35 ± 0.05	bcde
	3	1.53 ± 0.27	1.24 ± 0.06	0.76 ± 0.14	0.52 ± 0.26	bce
	4	1.76 ± 0.23	1.49 ± 0.04	1.23 ± 0.20	0.93 ± 0.19	bce
IL1α positive area (%)	0	1.54 ± 0.08	1.59 ± 0.05	1.64 ± 0.08	1.49 ± 0.36	–
	1	8.64 ± 0.35	7.85 ± 0.86	10.77 ± 1.32	14.51 ± 0.74	cdef
	2	6.42 ± 1.03	5.16 ± 0.89	8.79 ± 1.39	15.37 ± 1.25	cdef
	3	5.71 ± 0.39	4.86 ± 0.57	7.69 ± 0.22	15.19 ± 0.64	bcdef
	4	4.92 ± 0.14	1.66 ± 0.11	5.50 ± 0.93	10.48 ± 0.99	acdef
PPARγ positive area (%)	0	0.00 ± 0.00	0.00 ± 0.00	0.00 ± 0.00	0.00 ± 0.00	–
	1	14.50 ± 1.43	15.69 ± 1.24	12.51 ± 0.64	9.96 ± 1.35	ce
	2	13.14 ± 0.92	14.80 ± 1.27	9.86 ± 1.38	6.77 ± 0.30	bcdef
	3	8.45 ± 2.16	9.40 ± 1.43	7.30 ± 0.82	6.85 ± 0.38	–
	4	0.69 ± 0.11	0.27 ± 0.11	0.65 ± 0.37	0.00 ± 0.00	cf
IL10 positive area (%)	0	0.24 ± 0.01	0.23 ± 0.03	0.23 ± 0.02	0.24 ± 0.09	–
	1	1.31 ± 0.33	3.67 ± 0.75	1.17 ± 0.22	0.15 ± 0.05	ade
	2	5.25 ± 0.94	5.91 ± 1.09	3.65 ± 0.88	2.47 ± 0.30	ce
	3	2.95 ± 0.55	3.89 ± 0.56	1.81 ± 0.56	0.97 ± 0.31	cde
	4	0.50 ± 0.09	0.55 ± 0.33	0.33 ± 0.09	0.65 ± 0.31	–

EA, electroacupuncture; BMSCs, bone mesenchymal stem cells; PBS, phosphate-buffered saline; SD, standard deviation; *p* ≤ 0.05, post-hoc analysis with Bonferroni correction. *p* < 0.05 and statistically significant difference between groups: a, EA vs. EA + BMSCs; b, EA vs. BMSCs; c, EA vs. PBS; d, EA + BMSCs vs. BMSCs; e, EA + BMSCs vs. PBS; f, BMSCs vs. PBS.

Immunohistochemical staining results for inflammatory cytokines revealed that the percentage of positively stained areas for IL-1α and PPARγ in all groups significantly increased and peaked within the first week post-surgery, followed by a gradual decline from weeks 2 to 4 (Figures 8B,C). In contrast, the percentage of positively stained areas for IL-10 significantly increased during weeks 1–2, peaking at week 2, and then gradually decreased to normal levels by weeks 3–4 (Figure 8D). Throughout the postoperative period, the levels of the pro-inflammatory cytokine IL-1α in the BMSCs group were consistently lower than those in the control group (Figure 8B; *p* = 0.005, 0.001, <0.001, and <0.001). However, they still remained notably higher compared to the EA + BMSCs group, which underwent electroacupuncture treatment (Figure 8B; *p* = 0.023, 0.029, 0.001, and 0.001). Regarding anti-inflammatory cytokines, in comparison with the BMSCs group, the EA + BMSCs group showed a notably higher expression of PPARγ at week 2 (Figure 8C; *p* = 0.003). Moreover, at weeks 1 and 3, the IL-10 expression in EA + BMSCs group was significantly elevated (Figure 8D; *p* = 0.001 and 0.006). These results indicate that EA enhances the regenerative microenvironment post-BMSCs transplantation, thereby boosting its therapeutic efficacy (Table 5).

### 3.3 Sciatic nerve function index (SFI) for assessing motor function recovery

**Figure 9:**
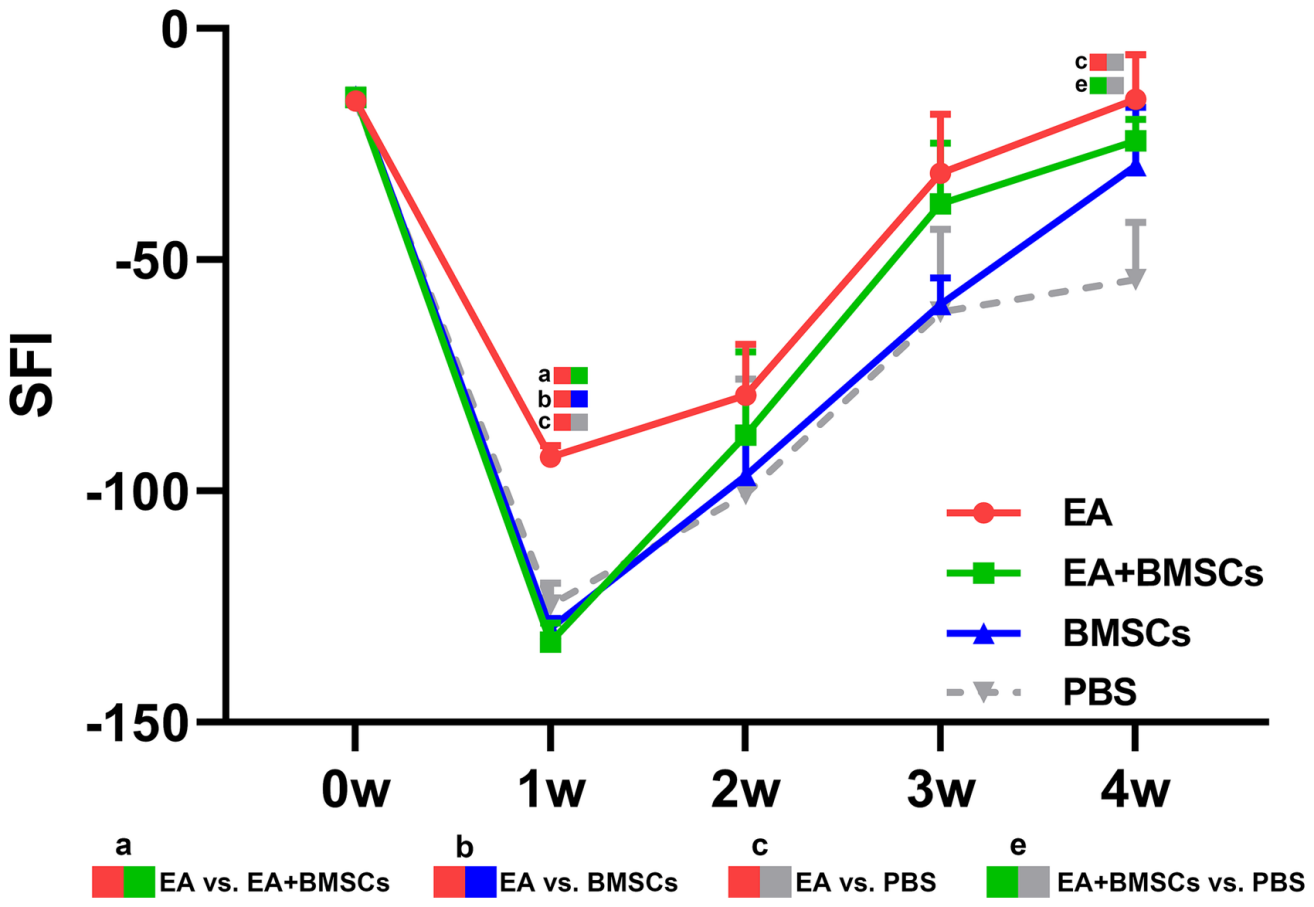
Comparison of the SFI among the four subgroups post-surgery. At week 1 post-surgery, the SFI was impaired in all groups, yet the EA group (red) exhibited the fastest recovery rate. From weeks 2 to 4, the SFI recovery rate in the EA group (red) surpassed that of the other groups, indicating that electroacupuncture (EA) treatment was the most effective in restoring nerve function. Table 6 shows the specific statistical analysis. W: week; SFI, sciatic nerve function index; EA, electroacupuncture; BMSCs, bone mesenchymal stem cells; PBS, phosphate-buffered saline.

The restoration of motor function in injured rats was gauged through the SFI. The SFI values of injured rats declined sharply within the first week post-surgery and gradually recovered from weeks 2 to 4 (Figure 9). At weeks 3 and 4 post-surgery, the EA group showed the greatest SFI levels, with the EA + BMSCs group ranking second. By week 4, SFI values ranked as follows: EA group > EA + BMSCs group > BMSCs group > PBS group. These findings indicate that injured nerves treated with EA achieved faster functional recovery post-surgery (Table 6).

**Table 6:** SFI values in EA, EA + BMSCs, BMSCs, and PBS (mean ± SD).

Time points (weeks)	EA	EA + BMSCs	BMSCs	PBS	*p* < 0.05
0	−15.67 ± 1.53	−15.00 ± 1.73	−14.67 ± 1.15	−15.67 ± 2.08	–
1	−92.67 ± 2.52	−132.67 ± 4.16	−129.67 ± 2.08	−124.67 ± 4.73	abc
2	−79.33 ± 11.02	−88.00 ± 18.08	−96.67 ± 9.87	−100.67 ± 24.85	–
3	−31.33 ± 12.74	−38.00 ± 13.23	−59.67 ± 5.69	−61.33 ± 17.95	–
4	−15.33 ± 9.61	−24.33 ± 4.62	−29.67 ± 12.66	−54.33 ± 12.42	ce

SFI, sciatic functional index; EA, electroacupuncture; BMSCs, bone mesenchymal stem cells; PBS, phosphate-buffered saline; SD, standard deviation; *p* ≤ 0.05, post-hoc analysis with Bonferroni correction. *p* < 0.05 and statistically significant difference between groups: a, EA vs. EA + BMSCs; b, EA vs. BMSCs; c, EA vs. PBS; e, EA + BMSCs vs. PBS.

### 3.4 Correlation of MRI with histological and functional outcomes

The correlations between MRI parameters of the injured sciatic nerve and histological and functional outcomes are presented in Supplementary Figure 1. Among three IVIM parameters, the nerve perfusion fraction (f) exhibited the strongest correlation with histological and functional outcomes (*r* = 0.70 to 0.94, all *p* < 0.001), outperforming other IVIM metrics.

## 4 Discussion

In a rat sciatic nerve crush injury model, we found that electroacupuncture (EA) treatment improved the regenerative microenvironment following stem cell transplantation. EA is more effective than bone mesenchymal stem cells (BMSCs) treatment alone in promoting myelin and axonal regeneration by reducing inflammatory responses and enhancing local microcirculation. Using intravoxel incoherent motion (IVIM) imaging, longitudinal changes in local microcirculation during the healing phase of peripheral nerve damage can be quantitatively assessed.

Studies have shown that injury to myelin and axons triggers pro-inflammatory cytokine release and peripheral tissue edema (26, 27). In addition, our findings show that microcirculatory perfusion is reduced after nerve injury, suggesting that excessive inflammatory response and ischemia may impair the regenerative microenvironment. To date, stem cells have been widely used to promote angiogenesis, modulate immune responses, and support nerve regeneration (28, 29). However, our investigation demonstrated that the therapeutic efficacy of stem cells was inferior to that of EA. Specifically, during the early post-injury phase (week 1), rats in the BMSCs group had a slower reduction of nerve edema, higher expression of pro-inflammatory cytokines, and a substantially larger area of myelin debris when contrasted with those in the EA group.

The suboptimal efficacy of stem cells therapy may be associated with immune rejection responses. When the immune system is minimally active, stem cells may provoke an immune reaction, leading to elevated inflammatory cytokine levels, enhanced infiltration of leukocytes and macrophages, and elevated levels of matrix metalloproteinases and oxidative stress (30, 31). Furthermore, the therapeutic effect of stem cells may be related to their directed differentiation and paracrine function. Research indicates that only a small proportion of transplanted cells survive and differentiate into Schwann cells during the transplantation process (32). However, this study did not track the transplanted BMSCs, making it impossible to determine the extent of their integration into host tissue or to assess their paracrine activity. Future studies should utilize techniques such as labeled cell imaging or fate-mapping to further elucidate the survival status and functional mechanisms of BMSCs during nerve repair.

Notably, this study found that EA + BMSCs combination helped alleviate edema following transplantation. T2 mapping showed that during the early injury phase (weeks 1–2), T2 values in the EA and EA + BMSCs groups recovered faster than in the BMSC group. Since T2 hyperintensity reflects edema caused by inflammatory mediators (33), this suggests EA effectively reduces inflammation-related edema post-transplantation. Additionally, our research findings showed that the levels of anti-inflammatory markers such as PPARγ and IL-10 in the EA + BMSCs group were higher than those in the groups treated with either method alone, suggesting a synergistic anti-inflammatory effect (Figures 8C,D). These findings are consistent with those of Wang et al. (34). This may be attributed to the ability of electroacupuncture to improve the local microenvironment and blood flow, thereby enhancing the survival of BMSCs and promoting the secretion of anti-inflammatory factors, ultimately leading to coordinated modulation of the inflammatory response and facilitation of nerve tissue repair (34).

In this study, EA demonstrated superior therapeutic effects on peripheral nerve injury, outperforming BMSCs in promoting myelin regeneration, functional recovery, and vascular repair. By week 4, the regenerated myelin area and SFI in the EA group were superior to those in the BMSCs group. These effects may be attributed to EA’s anti-inflammatory properties, as the EA group exhibited lower IL-1α and higher PPARγ and IL-10 expression from weeks 1 to 3. EA accelerated the clearance of myelin debris as well. Its anti-inflammatory effects may have reduced inflammatory cell infiltration and Schwann cell damage, creating a favorable microenvironment for regeneration (35). In addition, T2WI indicated that EA was more effective in alleviating muscle edema, which may help reduce pain and improve motor function (36, 37). Notably, expression of the endothelial marker CD31 was significantly higher in the EA group than in the BMSCs group, indicating enhanced angiogenesis. Improved blood perfusion may facilitate nutrient delivery and waste removal, further supporting tissue repair. Overall, EA outperformed BMSCs in multiple aspects, highlighting its superiority in neural repair.

Moreover, we observed that EA alone outperformed the EA + BMSCs combination in terms of myelin regeneration and SFI. This may be due to potential transplantation rejection responses or sterile inflammation induced by BMSCs; furthermore, the combination treatment might cause excessive tissue stimulation, thereby disrupting local microenvironmental homeostasis and slowing the nerve repair process. Additionally, during combination therapy, the multipotent differentiation capacity of BMSCs may lead to competition among differentiation pathways. Specifically, while most BMSCs may differentiate into target cells such as Schwann cells, a subset might instead differentiate into non-neural lineage cells such as glial cells, fibroblasts, or smooth muscle cells. This divergence in differentiation pathways may compromise the overall efficiency of nerve regeneration.

In this study, IVIM was used to evaluate changes in nerve perfusion. The perfusion fraction (f) serve as a sensitive and non-invasive indicator for assessing neurovascular injury and recovery (14, 15). Our findings suggest that EA promotes nerve regeneration by enhancing perfusion. EA has been proven to improve blood supply to peripheral nerves, delivering oxygen and nutrients essential for axonal growth (11). Moreover, during nerve regeneration, enhanced axonal transport—the improved capacity for intra-neuronal material transport—effectively meets the material demands of regenerating axons (38). Thus, increased nerve perfusion may result from the combined effects of improved blood supply and enhanced axonal transport. Notably, EA increased blood perfusion in muscles adjacent to the injured nerve as well. A similar study by Kubota et al. reported increased muscle blood volume following EA stimulation at ST36 and ST38 acupoints (39). Improved muscle perfusion may help reduce edema and alleviate pain, thereby facilitating spontaneous motor activity and accelerating functional recovery (40, 41). In summary, EA enhances perfusion in both nerves and surrounding muscles, creating a more favorable microenvironment for nerve regeneration and functional improvement.

Quantitative IVIM parameters, such as the perfusion fraction (f), can be further compared with histological findings to provide a more comprehensive understanding of nerve perfusion recovery. In this study, the injured nerve’s *f* value showed a rapid decline by week 1, likely attributable to microcirculatory impairment mediated by acute inflammation. Following PNI, monocytes migrate from the bloodstream to the injury site, where they differentiate into macrophages and secrete pro-inflammatory cytokines (42). These cytokines induce damage to vascular endothelial cells and contribute to tissue edema. Immunohistochemical staining results from this study corroborated that IL-1α expression peaked at week 1, coinciding with a robust inflammatory response that led to tissue edema. The resultant increase in edematous pressure may compress capillaries, reducing effective perfusion volume (43). Concurrently, heightened vascular permeability induced by inflammatory cytokines can cause plasma extravasation, further diminishing capillary blood volume and consequently lowering the *f* value (44).

From weeks 2 to 4, the perfusion fraction (f) of the injured nerve gradually increased. In comparison with the standalone BMSCs group and the PBS group, the EA group showed a more rapid increase in *f* values, indicating that EA treatment was the most effective in promoting nerve perfusion recovery. In our study, correlation analysis showed strong inverse relationships between f values and IL-1α (*r* = −0.90, *p* < 0.001) as well as PPARγ (*r* = −0.70, *p* < 0.001), suggesting that EA enhances nerve perfusion by modulating the inflammatory microenvironment. The process likely relies on the shift from a pro-inflammatory to an anti-inflammatory state, where EA boosts PPARγ expression through the STAT6/PPARγ signaling cascade (42, 45). This transition drives the conversion of pro-inflammatory M1 macrophages to anti-inflammatory M2 macrophages (42, 45). Concurrently, angiogenesis is activated, with increased expression of growth factors such as VEGF, facilitating the reconstruction of the capillary network (46). This results in an expanded microcirculatory perfusion volume, reflected by the rapid recovery of f values.

Among the three IVIM parameters, the perfusion fraction (f) exhibited a gradual recovery from weeks 2 to 4 following nerve injury, whereas the true diffusion coefficient (D) and pseudo-diffusion coefficient (D*) showed no significant differences. Klauß et al. reported similar findings in their study on steroid treatment for autoimmune pancreatitis, observing a significant increase in the f value during treatment, while D and D* values showed no notable changes (47). Several reasons may explain this. First, D value primarily reflects the free diffusion of water molecules within tissue (48). However, in nerve tissue, the highly oriented fiber structure restricts transverse diffusion, as water molecules predominantly diffuse along the axonal direction (13). Thus, D value may be less sensitive to structural changes. Second, D* value reflects microcirculatory blood flow velocity (48). Given the sparse capillary network in peripheral nerves, D* value may be unstable in such tissues. Additionally, this study found poor interobserver reproducibility for both D and D* measurements. Overall, D and D* values may be less suitable as reliable quantitative biomarkers in this context.

Our study has several limitations. First, we used the sciatic nerve function index (SFI) to assess motor function recovery in animals. Future studies could incorporate tests such as the thermal withdrawal latency test to more comprehensively assess sensory nerve recovery. Second, although this study measured inflammatory markers such as IL-1α, PPARγ, and IL-10, the limited volume of injured nerve tissue precluded direct phenotyping of M1/M2 macrophages. Third, the clinical application of stem cell therapy still faces certain challenges, making the EA + BMSCs combination difficult to directly translate to human treatment. However, our research findings suggests that as a safe and feasible approach, EA may have greater potential for clinical translation. Fourth, although the rats in the EA group showed near-complete functional recovery by week 4, future studies could extend the observation period to weeks 6, 8, and 12 to better evaluate the long-term effects of EA and EA + BMSCs combination. Finally, future studies should include a restraint-only group (without EA stimulation) to more precisely evaluate the specific effects of electroacupuncture.

## 5 Conclusion

In summary, our study demonstrates that electroacupuncture (EA) serves as an effective therapeutic approach for peripheral nerve injury repair, outperforming both BMSCs alone and the EA + BMSCs combination, while maintaining safety and feasibility in clinical practice. EA not only improves the regenerative microenvironment following stem cell transplantation but also promotes nerve regeneration and functional recovery by reducing inflammation and improving blood perfusion. Furthermore, the perfusion fraction (f) derived from intravoxel incoherent motion (IVIM) imaging serves as a dynamic and non-invasive biomarker for monitoring perfusion changes and evaluating the effectiveness of nerve recovery.

## Data Availability

The datasets presented in this study can be found in online repositories. The names of the repository/repositories and accession number(s) can be found below: zenodo repository. The DOI link is: 10.5281/zenodo.16200121.

## Supplementary material

SUPPLEMENTARY FIGURE 1 Heatmap and scatter plots showing the correlations between IVIM-MRI metrics and histological/functional outcomes. **(A)** This figure illustrates the Pearson correlation coefficients between IVIM-MRI metrics and histological/functional outcomes. The *Y*-axis represents MRI metrics; the *X*-axis represents histological/functional indicators. Colors denote the direction of correlation, with red indicating positive correlation and blue indicating negative correlation; the intensity of the color and the size of the circles reflect the strength of the correlation. Asterisks denote levels of statistical significance. **(B–G)** Scatter plots showing the correlations between perfusion fraction (f) and histological/functional outcomes. Compared with D and D*, f showed a higher correlation with histological/functional outcomes. f, perfusion fraction; D, diffusion coefficient; D*, pseudo-diffusion coefficient; SFI, sciatic nerve function index; *, *p* < 0.05; **, *p* < 0.01; ***, *p* < 0.001.
